# Skilled reaching test for shoulder function assessment in a rat model of rotator cuff tear: a pilot study

**DOI:** 10.1186/s12891-024-07624-6

**Published:** 2024-06-28

**Authors:** Yang Liu, Sai-Chuen Fu, Shi-Yi Yao, Patrick Shu-Hang Yung

**Affiliations:** 1 Department of Bone and Joint Surgery, Shenzhen People’s Hospital, Shenzhen , Guangdong, 518020 China; 2 Department of Orthopaedics and Traumatology, Faculty of Medicine, Lui Che Woo Clinical Science Building, The Chinese University of Hong Kong, Prince of Wales Hospital, 5/F, RoomShatinHong Kong SAR, NT 74029 China; 3 The Second Clinical Medical College, Jinan University, Shenzhen, Guangdong China; 4 The First Affiliated Hospital, Southern University of Science and Technology, Shenzhen, Guangdong China; 5 Institute of Innovative Medicine, The Chinese University of Hong Kong, Hong Kong SAR, China

**Keywords:** Skilled reaching test, Gait analysis, Rotator cuff repair, Shoulder function, Animal model

## Abstract

**Background:**

Functional assessments are crucial to evaluate treatment outcomes in clinical and animal studies on rotator cuff injuries. While gait analysis is commonly used to assess animal models of rotator cuff tears, it is less relevant for human patients as the human shoulder is typically assessed in a non-weight-bearing condition. The present study introduces the skilled reaching test as a shoulder functional assessment tool for rats, which allows for evaluation without weight bearing.

**Methods:**

In the control group, 8 male Sprague–Dawley rats received rotator cuff tear surgery without repair. In the rotator cuff repair group, 20 rats received rotator cuff repair at 4 weeks post rotator cuff tear. For the skilled reaching test, rats were trained to extend their forelimbs to fetch food pellets, and the number of trials, number of attempts and the success rate were recorded. The gait analysis and skilled reaching test were performed at baseline, 4 weeks post-tear, 1, 2, 4, and 8 weeks post-repair. The repeated measures analysis of variance was used to evaluate the effects of time on the shoulder function. The significance level was set at 0.05.

**Results:**

The skilled reaching test required 216 h to conduct, while the gait analysis took 44 h. In the rotator cuff repair group, gait performance significantly deteriorated at 1 week post-repair and restored to 4 weeks post-tear levels at 4 weeks post-repair. Regarding the skilled reaching test, the number of attempts, number of trials and the success rate decreased at 1 week post-repair. Subsequently, there was a brief rebound in performance observed at 2 weeks post-repair, followed by a continued decline in the number of attempts and trials. By 8 weeks post-repair, only the success rate had restored to levels similar to those observed at 4 weeks post-tear.

**Conclusion:**

The skilled reaching test can detect functional deficiencies following rotator cuff tear and repair, while it requires high time and labour costs.

**Supplementary Information:**

The online version contains supplementary material available at 10.1186/s12891-024-07624-6.

## Background

The incidence of rotator cuff (RC) tears is high and tends to increase with age. A study reported that 12.8% of individuals in their 50 s had RC tears, while the prevalence increased to 25.6% among those in their 60 s [1]. Among individuals with RC tears, approximately 34.6% may experience shoulder pain and dysfunction [1–4]. Consequently, patients commonly seek interventions aimed at alleviating pain and restoring shoulder function.

In animal studies, shoulder function is a primary outcome measure for RC tears, typically evaluated using gait analysis, open field, staircase, range of motion, and running duration [5]. The walking gait analysis is the most used assessment. It was reported that the contact intensity and pawprint area decreased by 40% at 1-week post RC tears [6]. However, the gait analysis could only assess the back-and-forth movements under a weight-loading condition, and the open field test could not precisely capture the forelimb performance but interfered with the other parts of the body. Contrarily, human shoulders seldom bear body weight and usually move in a larger range of motion towards multiple directions. Therefore, there is a need for a new functional assessment that focuses on shoulder function with clinical relevance.

The skilled reaching test was originally used in neurological studies to investigate motor control in brain injuries [7–9]. In this test, rats are trained to fetch food pellets with a forelimb, involving paw extension to the head level, similar to humans [10]. Cineradiography/video X-ray analysis has shown that forelimb advancement involves some shoulder joint extension, indicating that the skilled reaching test is applicable for revealing shoulder function [11]. To the best of our knowledge, we are the first to adapt the skilled reaching test to the field of orthopaedics to assess shoulder function after RC tears in animals.

Therefore, a non-weight-bearing assessment of rat shoulder function is needed, and the shoulder function following RC repair needs to be further investigated using traditional gait analysis and the newly introduced skilled reaching test. This study aims to assess the rat model’s shoulder function with the skilled reaching test. The hypothesis was that the skilled reaching test can detect impairment of shoulder function in the rat model.

## Methods

All animal studies were done in compliance with the regulations and guidelines of the Animal Experimentation Ethics Committee at the Chinese University of Hong Kong and conducted according to the ARRIVE guidelines 2.0. All animals were ordered from the Laboratory Animal Services Centre at the Chinese University of Hong Kong and held in the animal house at the Prince of Wales Hospital. Rats were maintained under standardized conditions with a 12:12 h light–dark cycle starting at 8:00 AM and a room temperature of 25 °C.

**Fig. 1:**
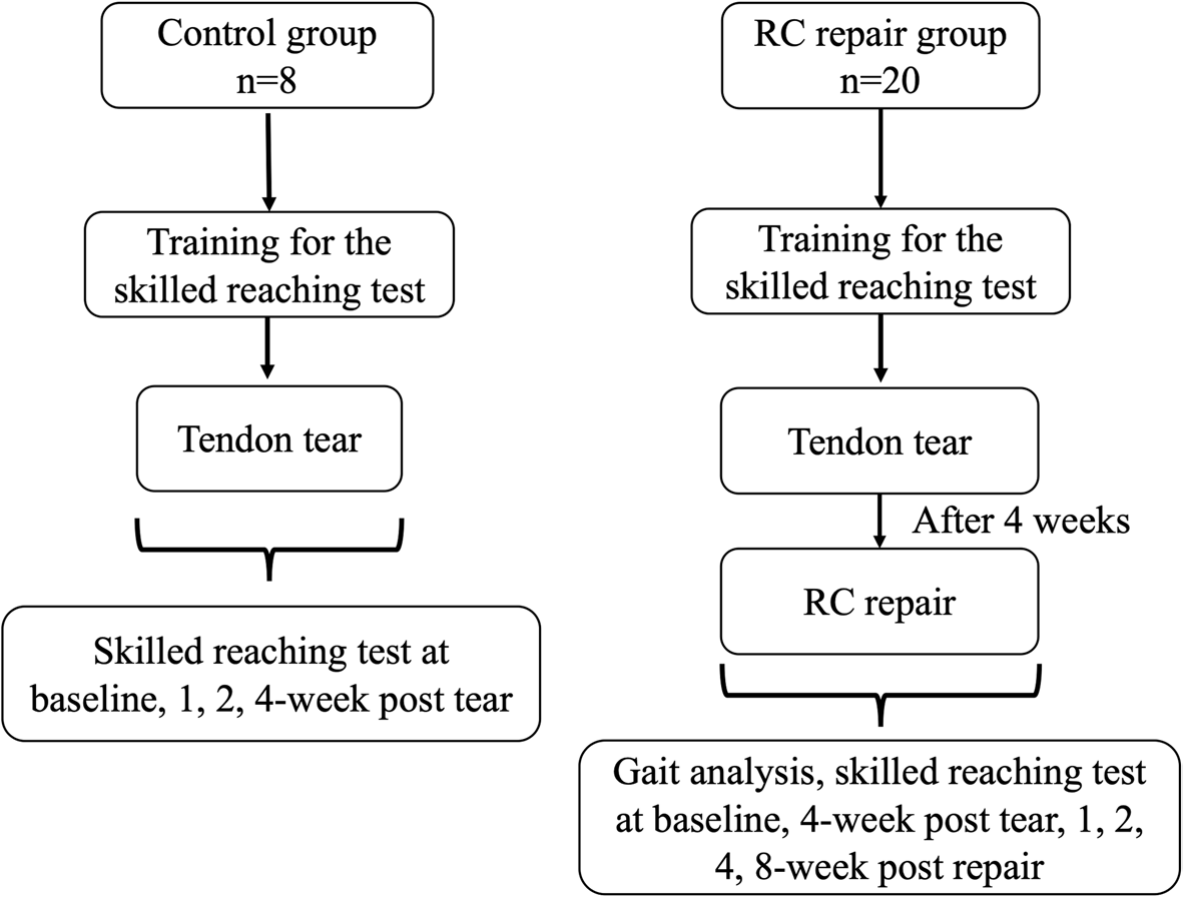
Study design

A total of 28 male Sprague–Dawley rats weighing 400 to 450 g were randomly assigned to two groups: RC repair (*n* = 20), and a control group (*n *= 8). In the RC repair group, surgery was conducted four weeks after the tendon tear, while the control group underwent supraspinatus tendon tear without any further surgical intervention. The study design is shown in Fig. 1.

### Surgical procedures

#### Rotator cuff tear

All rats underwent a standardised surgical procedure on the supraspinatus tendon by the same operator (Yang LIU) [12]. Prior to the operation, a subcutaneous injection of buprenorphine (0.05 mg/kg) was given for pain relief. Anaesthesia was induced with an intraperitoneal injection of ketamine (75 mg/kg) and xylazine (10 mg/kg). A 1.5 cm skin incision was made longitudinally above the acromion in the supine position, and the deltoid muscle was split along the direction of muscle fibres to expose the supraspinatus tendon. The tendon was then isolated and transected from the bony insertion. After the surgery, rats were individually housed for 24 h and allowed free cage activities with fed ad libitum feeding. Buprenorphine (0.05 mg/kg) was administered subcutaneously every 12 h for 72 h to manage postoperative pain.

#### Rotator cuff repair

Four weeks after the supraspinatus tendon was transected, scar tissues were excised, and the supraspinatus tendon was mobilised to approximate the greater tuberosity. The distal end of the tendon was stitched using 5–0 Vicryl ™ suture (Ethicon, Inc, New Jersey, USA) with the Modified Mason-Allen stitches [13]. Then the tendon was fixed onto the footprint with the sutures.

### Functional assessments

All functional assessments were conducted in the morning to minimize the impact of circadian rhythms on animal behaviour. Rats were trained for the skilled reaching test before receiving any surgical intervention.

#### Skilled reaching test

**Fig. 2:**
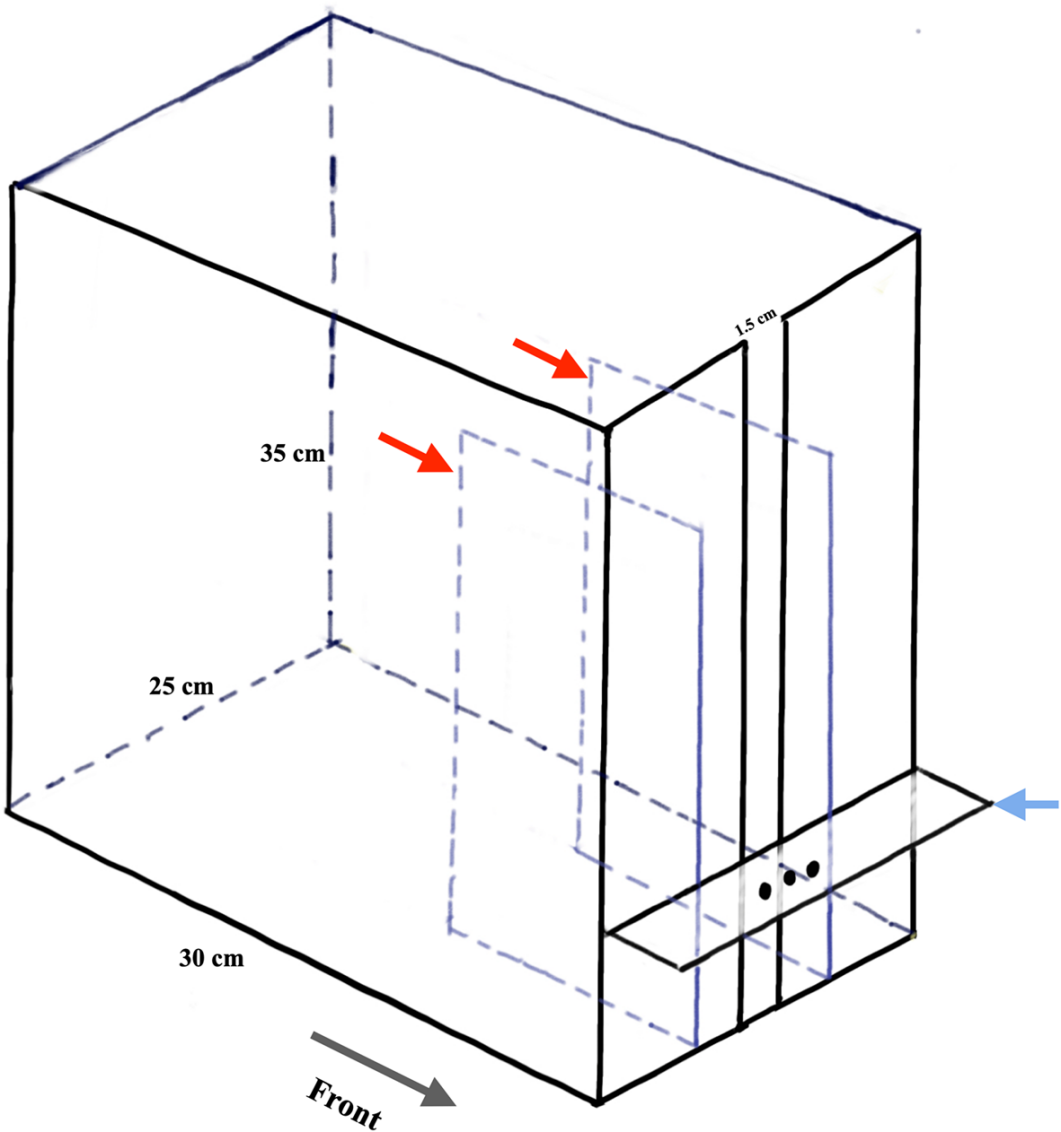
A schematic shows the custom-made transparent acrylic chamber for the skilled reaching test. The size of the chamber was 30 × 25 × 35 cm. A 1.5 cm wide slot was made at the middle front for the rat to reach for food pellets. Two thin plates spaced 10 cm apart, stand vertically on both sides of the slot to control the position of the rat’s body (red arrows). A table, at 3 cm above the floor, was attached outside the slot (blue arrow) with three indentations (three black dots at the centre of the table) to hold the food pellet. Each indentation was 0.5 mm deep, Ø 3 mm, and 10 mm in between. The appearance of the chamber can be seen in the Additional File 1

According to a previous study [14], the skilled reaching test was performed in a custom-made chamber, as shown in Fig. 2.

To maintain body weight and deprivation, food supply for rats was restricted to 0.05 g/g body weight/ day.

Rats were trained for 30 min every day for two weeks. Initially, five pellets were placed on the table, close enough for the rats to reach with tongue. Gradually, the pellets were moved further away from the slot to encourage the rat to fetch them with forepaw. The paw that was used more than 70% of the time was identified as the dominant side.

During the training section, each rat each rat had a 20-min session to retrieve as many pellets as desired. A digital camera (Sony MX 100, Sony Corporation. Tokyo, Japan) was set beside the chamber to record movements during the test. Any motion of the forelimb intended to reach the pellet is defined as an attempt regardless the result (Events 1–5 in Table 1). Any motion of the rat that moved the pellet and required the intervention of researchers to start over a test is defined as a trail (Events 3–6 in Table 1) [15]. Event 6 was defined as the pellet on the table was taken with tongue with or without the help of a limb.

**Table 1:** Evaluating system of the skilled reaching test

Event	Symbol	Meaning	Definition of attempts	Definition of trials
1		Forelimb advanced but did not touch the pellet	Events 1–5 were counted as an attempt	
2		Forelimb advanced and touched the pellet. The pellet either stayed in the indentation or moved but was within reach		
3		The pellet was reached by a paw and eaten		Events 3–6 were counted as a trial
4		The pellet was knocked off the table		
5		The pellet was touched by a paw but moved out of reach		
6		Pellet on the table was obtained by tongue		
7		No forelimb advanced		

The number of attempts, trials, and success rate were reported as outcome measurements. The success rate was calculated as follows [16].

$$Total\;success\;rate=100\%\times\frac{Number\;of\;success\;reaches}{Number\;of\;trials}$$

Inclusion criteria: after the 2-week training section, and before any surgical treatment, rats that finished 20 trials in 20 min with a success rate higher than 50% were included. The results recorded in the inclusion test is also presented as the baseline performance. Rats that failed to meet the inclusion criteria were kept in each group, receiving the same surgical treatment as those passed the criteria. The video clips of each rat’s performance were assessed and documented by the same reviewer (Yang LIU) using the evaluating system presented in Table 1, with this process being conducted three times. Any inconsistencies in the records were cross-checked against the video clip.

#### Gait analysis

The downhill walking gait analysis was performed using the CatWalk ™ XT 9.0 (Noldus, Netherlands), following the protocol from a previous study [17]. The walkway’s starting point was tilted at a 10° angle relative to the horizontal plane allowing the rats to traverse the entire course voluntarily.

For each rat, 2 to 4 compliant walks were included for analysis, and speed variation was limited to within 30%. Images of pawprints were automatically detected and recorded by a digital camera below the walkway. The maximum intensity, pawprint area, stride length and swing duration of the forelimbs were automatically calculated by built-in software. All gait parameters were normalised as a ratio between the operated and contralateral sides for each rat.

### Statistics

The statistical analysis for this study was conducted using SPSS (Version 25, IBM Corp, Armonk, N.Y., USA). The distribution of normality and the equality of variance was tested using the Shapiro–Wilk and Levene’s tests, respectively. The effect of time after repair on the gait and skilled reaching performance was analysed using the repeated measures analysis of variance or Friedman test. To account for multiple comparisons, the significant level was adjusted with Bonferroni. A *p*-value of less than 0.05 was considered statistically significant.

## Results

All rats survived the surgery; no wound complication was noticed.

### The skilled reaching test

**Fig. 3:**
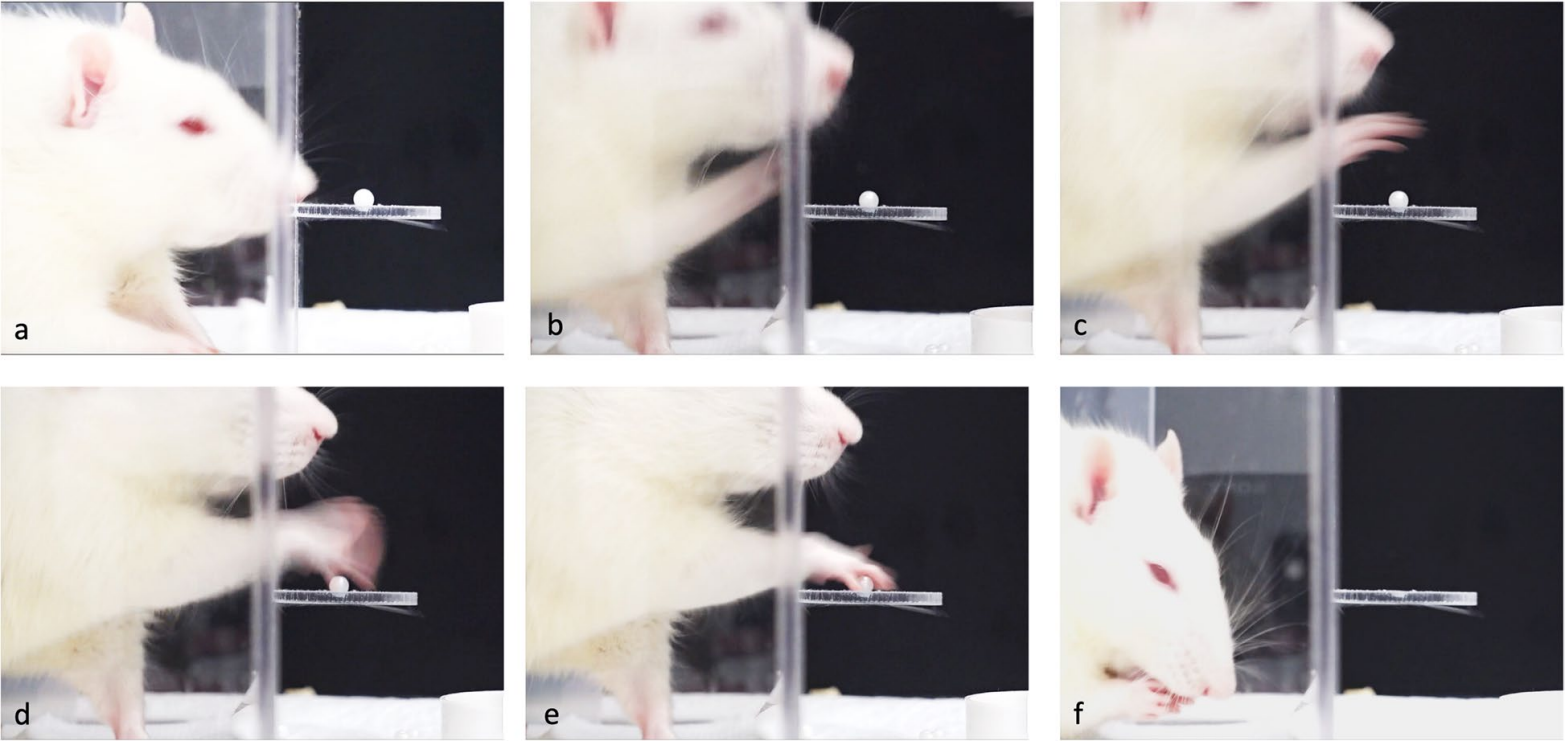
Pictures of the detailed movements of a rat during the process of skilled reaching. **a** Starting position. **b** Forward extension of the forelimb and lifting the elbow with pronation of the forelimb. **c** Advancing of the forelimb till the paw reached over the pellet. **d** Further pronation of the forelimb, opening the digits and lowering the paw to reach the pellet. **e** Grasping the pellet. **f** Withdrawal and supination of the forelimb to place the food in the mouth

After training, 3 out of 8 rats in the control group, 7 out of 20 rats in the RC repair group met the inclusion criteria. Acclimation and training of the 28 rats costed 216 h. The rats were able to stretch out their dominant forelimb through a narrow slot and grasp the pellet with a paw, then retract the paw, and supinate the forelimb to serve the pellet to mouth. The detailed movements are shown in Fig. 3, and a video clip of a typical reaching performance of a rat in the RC repair group at 1 week post-repair is available in Additional file 1.

**Fig. 4:**
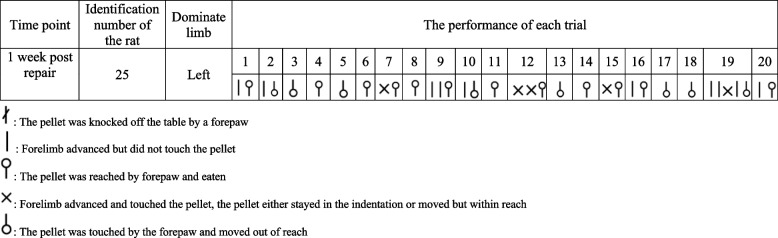
Example of the performance record of the skilled reaching test. Rat No. 25 made 35 attempts in 20 trials. Twelve successful reaches were made

Figure 4 demonstrates the records of a rat's performance in the skilled reaching test, utilizing the aforementioned symbols.

#### Control group

Table 2 provides the descriptive statistics of the control group.

**Table 2:** Number of trials, attempts, and success rate of the control group

Parameter	Rat ID	Baseline	Post tear		
			1 week	2 weeks	4 weeks
Number of Trials	Rat A26	20	20	20	17
	Rat A27	20	20	20	20
	Rat A28	20	20	20	20
Mean ± SD		20 ± 0	20 ± 0	20 ± 0	19 ± 1.7
Number of attempts	Rat A26	37	42	45	37
	Rat A27	37	35	40	41
	Rat A28	36	32	36	40
Mean ± SD		36.7 ± 0.6	36.3 ± 5.1	40.3 ± 4.5	39.3 ± 2.1
Success rate	Rat A26	55.0%	45.0%	45.0%	52.9%
	Rat A27	63.3%	30.0%	60.0%	55.0%
	Rat A28	53.3%	65.0%	50.0%	55.0%
Mean ± SD		57.2 ± 5.4%	46.7 ± 17.6%	51.7 ± 7.6%	54.3 ± 1.2%

The number of trials and attempts was not significantly affected by RC tear at any time. At 1 week post-tear, the average success rate decreased from 57.2% at baseline to 46.7%, and it gradually restored to 54.3% at 4 weeks post-tear.

#### RC repair group

The average number of trials, number of attempts and success rate declined at 1 week post-repair, recovered to the 4-week post-tear level at 2 weeks post-repair, and declined again at 4 weeks post-repair (Table 3). At 8 weeks post-repair, the average number of trials and attempts further declined while the average success rate restored to 65.6%.

**Table 3:** Number of trials, attempts and success rate of the rotator cuff repair group

Parameter	Rat ID	Baseline	4-week post tear	Post repair			
				1 week	2 weeks	4 weeks	8 weeks
Number of trials	22	20	20	0	20	20	8
	23	20	20	14	20	20	7
	25	20	20	20	20	20	20
	56	20	11	7	9	4	6
	57	20	20	15	20	17	11
	61	20	10	11	20	20	8
	70	20	20	9	20	20	15
Mean ± SD		20 ± 0	17.3 ± 4.6	10.9 ± 6.4	18.4 ± 4.2	17.3 ± 6	10.7 ± 5.1
Number of attempts	22	33	32	12	35	29	13
	23	34	35	42	37	35	13
	25	35	29	35	28	34	28
	56	47	36	29	28	6	24
	57	48	41	35	44	34	21
	61	37	15	24	35	31	12
	70	40	36	27	53	35	28
Mean ± SD		39.1 ± 6.1	32 ± 8.4	28.6 ± 9.4	37.1 ± 8.9	29.1 ± 10.4	19.9 ± 7.2#
Success rate	22	75%	75%	0%	55%	55%	63%
	23	80%	60%	50%	60%	45%	71%
	25	70%	75%	60%	80%	65%	65%
	56	80%	55%	43%	56%	25%	67%
	57	60%	10%	53%	65%	53%	82%
	61	90%	40%	45%	45%	60%	25%
	70	70%	70%	44%	50%	40%	87%
Mean ± SD		75 ± 9.6%	54.9 ± 23.5%	43 ± 20.4%	58.7 ± 11.4%	49 ± 13.5%	65.6 ± 20%

^#^: *p* < 0.05 compared to 4 weeks post-tear

Statistical analysis revealed a significant effect of time on these parameters (*p* = 0.000 for average number of trials, *p* = 0.006 for number of attempts, and *p* = 0.016 for success rate), indicating significant changes over the follow-up period. However, the differences between the 4 -week post-tear time point and other follow-up time points were not statistically significant, except for the number of attempts at 8 weeks post-repair, which was significantly lower compared to 4 weeks post-tear (*p* = 0.045).

**Fig. 5:**
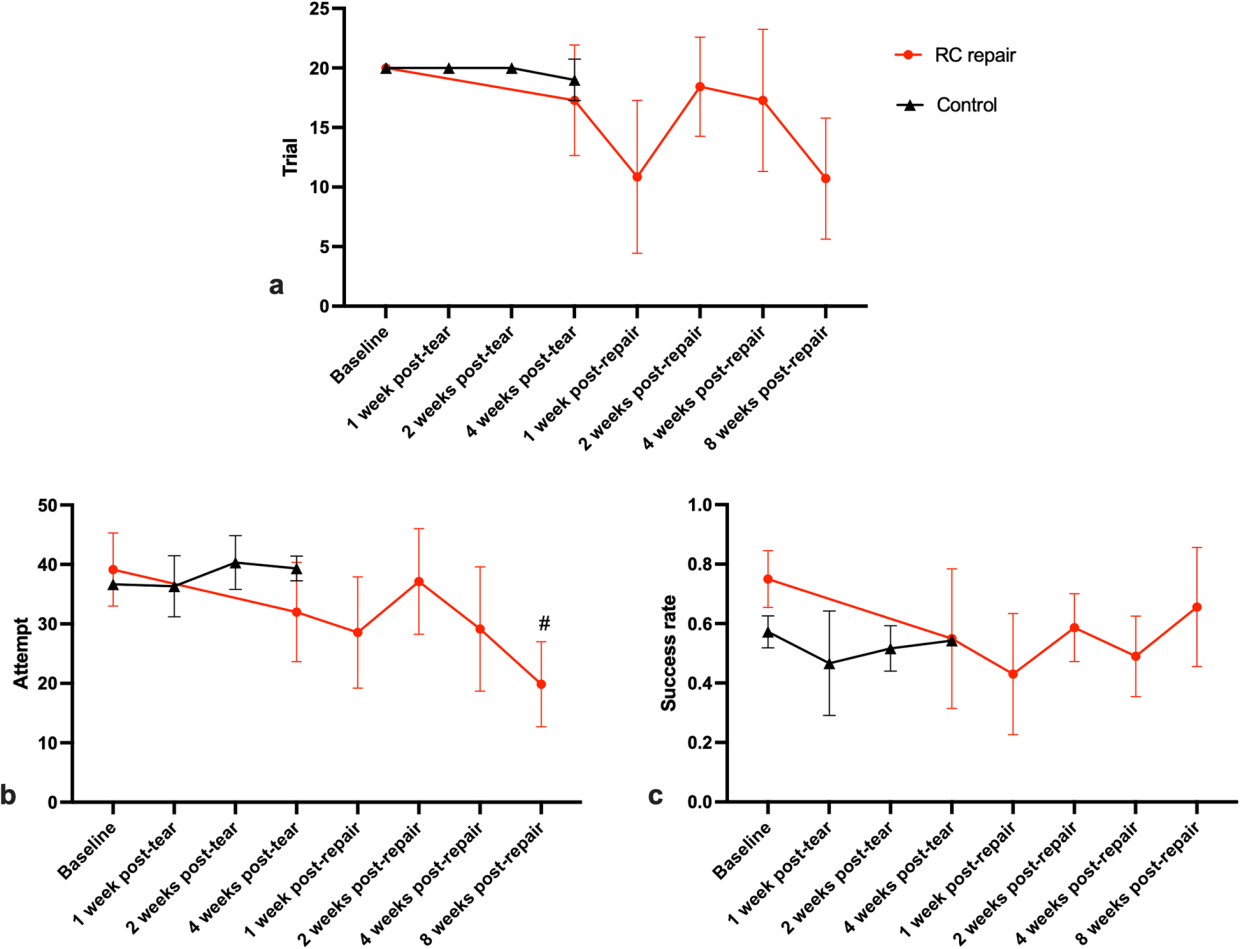
Results of the skilled reaching test. The number of trials, number of attempts and the success rate of the RC repair group reduced at 1 week post-repair and restored at 2 weeks post-repair. At 4 and 8 weeks post-repair, the average number of trials and attempts further declined. RC: rotator cuff. #: *p* < 0.05 compared to 4 weeks post-tear. Data are presented as mean ± SD

The trends of performance in the skilled reaching test through all time points are shown in Fig. 5.

### The gait analysis

This study investigated the gait performance of the 20 rats in the RC repair group, with a total of 14 h dedicated to acclimation and approximately 30 h for testing and data collection. The normality of distribution and the equality of variance were both assumed. There were significant decreases in the max intensity, print area, stride length and swing duration at 1 week post-repair (*p* = 0.002, *p* = 0.01, *p* = 0.005, *p* = 0.002 respectively) compared to those at 4 weeks post-tear. Both the max intensity and print area rebounded to the 4-week post-tear levels by 2 weeks post-repair, while the stride length and the swing time rebounded to 4-week post-tear levels by 4 weeks post-repair. All the gait parameters remained stable at 8 weeks post-repair.

The description is shown in Table 4.

**Table 4:** Gait analysis of the RC repair group (Mean ± SD)

	Baseline	4 weeks post-tear	1 week post-repair	2 weeks post-repair	4 weeks post-repair	8 weeks post-repair
Max Intensity	0.96 ± 0.08	0.99 ± 0.06	0.66 ± 0.32^**#**^	0.91 ± 0.1^**#**^	0.96 ± 0.1	0.97 ± 0.09
Print Area	1 ± 0.19	0.93 ± 0.29	0.41 ± 0.46^**#**^	0.59 ± 0.28^**#**^	0.8 ± 0.28	0.91 ± 0.18
Stride Length	0.97 ± 0.09	1.03 ± 0.08	0.63 ± 0.45^**#**^	0.93 ± 0.25	0.96 ± 0.11	1.03 ± 0.12
Swing Time	1.02 ± 0.11	1.16 ± 0.46	3.3 ± 2.15^**#**^	1.46 ± 0.42	1.25 ± 0.37	1.24 ± 0.33

Data are ratios between the operated and contralateral sides

*RC* Rotator cuff

^#^: *p* < 0.05 compared to 4 weeks post-tear

## Discussion

This pilot study introduces the skilled reaching test as a novel functional assessment tool for assessing rotator cuff tear and repair. The skilled reaching test can capture functional deficiencies for the RC repair model.

The study found that the skilled reaching test revealed a similar trend of functional recovery as found in the gait analysis, with function declining at 1 week post-repair and being restored at 2 weeks post-repair. The impairment in the shoulder function may be due to the surgical trauma and impaired rotator cuff, which compromised the enthusiasm and capability of the animals to use the injured shoulder.

At 2 weeks post-repair, all the skilled reaching and gait parameters promptly restored, indicating that most of the pain had subsided, and the animals' willingness to reach the food began to recover. However, the average number of attempts and number of trials continued declining at 4 and 8 weeks post-repair, indicating that the series of skilled reaching movements remain challenging for the animals.

### The skilled reaching test

The skilled reaching test is a more clinically relevant test than the gait analysis due to its non-weight bearing nature and the requirement for a broader range of motion during forward extension of the forelimb. Based on lateral X-ray measurements of the rat’s movement process provided in the references [11, 18], the degree of forward extension (defined as the angle between the scapular spine and the humerus axis in a lateral view) observed in the skilled reaching test was 37.4°, which was greater than the 18.8° recorded during the gait analysis test.

Furthermore, the skilled reaching test requires more intensive contraction and rotation of the rotator cuff compared to gait analysis. In the skilled reaching test, a rat must elevate its forelimb 3 cm above the ground, whereas during walking, the elevation required is less than 1 cm. This could account for the observation that the average number of attempts and number of trials remain low at 4 and 8 weeks post-repair.

However, the skilled reaching test is also time-consuming and exhibits high individual variability. Training and testing 20 rats for the skilled reaching test took 216 h, in contrast to 44 h for gait analysis. This duration is consistent with a previous study that expended 188 h on 13 rats in the skilled reaching test [19]. Additionally, only one-third of the rats in the current study satisfied the inclusion criteria, aligning with findings from previous research [20]. Factors such as the animal's training environment, food pellet, and individual behaviour may impact the effectiveness of the training. These challenges underscore the importance of enhancing the training and assessment efficiency of the skilled reaching test. Recent researches have introduced automated systems for training and assessment in skilled reaching, potentially increasing the test's efficiency [20–22]. However, the validity and reliability of these automated systems remain to be substantiated. Overall, although the skilled reaching test demonstrates potential as a more clinically pertinent measure of functional recovery post rotator cuff tear and repair, additional research is necessary to refine its efficiency and confirm its validity and reliability.

### The gait analysis

The gait analysis has limitations in its capacity to detect deficiencies in shoulder function following RC tear and repair, as it does not directly measure the load bearing on the injured limb. In this study, gait analysis only identified deficiencies within the initial 2-week period post-repair, consistent with findings from prior research [23].

Moreover, the rapid restoration of function may be attributed to the superior healing capacity observed in the rat model. Previous studies have noted intensive scar formation at the tendon stump-humeral head gap, which potentially accelerates the recovery of the gait [24–26].

In summary, while gait analysis remains a valuable tool for evaluating functional recovery post-RC tear and repair, its efficacy in identifying shoulder function deficiencies is limited. Contact force emerges as a more direct indicator of loading capacity, while the rat model’s enhanced healing capacity likely contributes to the swift functional restoration observed in this investigation.

### Kinematics of the glenohumeral joint

It is worth noting that although rats share similarities in shoulder anatomy and forelimb movements, particularly in reaching, with humans, there are significant differences in the kinematics of the glenohumeral joint [10, 18]. X-ray imaging studies have shown that when rats extend their forelimbs, movements of the elbow and scapula play a substantial role in forelimb advancement [11]. This phenomenon could be attributed to the sagittal plane positioning of the rat’s scapula, facilitating easy forward extension of the forelimbs, whereas the human scapula is positioned closer to the coronal plane. Furthermore, the subscapularis muscle and serratus anterior in rats exhibit greater muscularity, indicating active engagement of the scapula in forelimb motions. Consequently, it is speculated that in rats, the scapula, rather than the rotator cuff, predominantly governs reaching and walking movements. Thus, while the skilled reaching test and gait analysis conducted in rat models provide insight into overall shoulder function, they may not precisely target the glenohumeral joint. It is prudent to interpret the results of these tests cautiously concerning the function of the rotator cuff.

### Limitations

The current pilot study has several limitations. Firstly, the sample size was small, and further studies with larger sample sizes are necessary to validate the findings of this study. Secondly, the assessment of shoulder motions in the skilled reaching test was subjective, and the parameters were limited to count data. Future studies could benefit from incorporating kinematic measurements, such as the velocity and angle of the glenohumeral joint, to gain a more comprehensive understanding of how rotator cuff tendon injuries impact forelimb movement and contribute to missed reaches. Thirdly, the rat’s preference for food may have influenced the outcomes of the skilled reaching test. Future studies may want to consider controlling for this variable to ensure that the observed effects are specifically related to the rotator cuff tear and repair. Finally, the present study solely focused on evaluating the overall impact of rotator cuff tear and repair on shoulder function. The results do not specify which specific factors, such as pain, range of motion, muscle strength, or healing, influence shoulder function. Future investigations may need to delve into these individual factors to provide a more nuanced understanding of the outcomes.

## Conclusions

The pilot study introduces a novel non-weight-bearing assessment tool for assessing shoulder function in the rat rotator cuff tear model. The skilled reaching test can detect functional deficiencies following rotator cuff tear and repair. However, the use of the skilled reaching test is deemed less cost-effective due to low compliance, high variation, and time burden. To validate these findings and enhance the comprehension of the functional implications of RC tear and repair, further studies with larger sample sizes and objective measurements are needed.

### Supplementary Information

Additional file 1. Video clip of a rat’s skilled reaching test. A video clip of the reaching performance of a rat at 1 week post-repair shows the motions of shoulder in skilled reaching test. The score of each trial is also shown in the video.

## Data Availability

The datasets analysed during the current study are available from the corresponding author on reasonable request.

## Abbreviation

RC: Rotator cuff
